# Attention-Based Recurrent Neural Network for Plant Disease Classification

**DOI:** 10.3389/fpls.2020.601250

**Published:** 2020-12-14

**Authors:** Sue Han Lee, Hervé Goëau, Pierre Bonnet, Alexis Joly

**Affiliations:** ^1^Swinburne University of Technology Sarawak Campus, Kuching, Malaysia; ^2^AMAP, Univ Montpellier, CIRAD, CNRS, INRA, IRD, Montpellier, France; ^3^CIRAD, UMR AMAP, Montpellier, France; ^4^INRIA Sophia-Antipolis - ZENITH Team, LIRMM - UMR 5506, Montpellier, France

**Keywords:** plant disease classification, deep learning, recurrent neural network, automated visual crops analysis, precision agriculture technologies, crops monitoring, pests analysis, smart farming

## Abstract

Plant diseases have a significant impact on global food security and the world's agricultural economy. Their early detection and classification increase the chances of setting up effective control measures, which is why the search for automatic systems that allow this is of major interest to our society. Several recent studies have reported promising results in the classification of plant diseases from RGB images on the basis of Convolutional Neural Networks (CNN). These studies have been successfully experimented on a large number of crops and symptoms, and they have shown significant advantages in the support of human expertise. However, the CNN models still have limitations. In particular, CNN models do not necessarily focus on the visible parts affected by a plant disease to allow their classification, and they can sometimes take into account irrelevant backgrounds or healthy plant parts. In this paper, we therefore develop a new technique based on a Recurrent Neural Network (RNN) to automatically locate infected regions and extract relevant features for disease classification. We show experimentally that our RNN-based approach is more robust and has a greater ability to generalize to unseen infected crop species as well as to different plant disease domain images compared to classical CNN approaches. We also analyze the focus of attention as learned by our RNN and show that our approach is capable of accurately locating infectious diseases in plants. Our approach, which has been tested on a large number of plant species, should thus contribute to the development of more effective means of detecting and classifying crop pathogens in the near future.

## 1. Introduction

Plant diseases are a major threat to agricultural production, causing severe food recessions and affecting crop quality (Bhange and Hingoliwala, 2015). To detect plant diseases in crops, plant pathologists generally use molecular and serological methods or measurements of various parameters, such as morphological change, temperature change, change in transpiration rate, or volatile organic compound emission from infected plants (Fang et al., 2015). Although it is an effective means of controlling plant diseases, consulting experts is nonetheless a costly and time-consuming process, especially since it is not always easy to bring an expert in time before the disease spreads to the crops. In recent years, automated classification of plant diseases has been addressed by the computer vision community to compensate for the lack of human expertise. Researchers used deep learning techniques to automatically identify diseases in individual crops, such as banana (Selvaraj et al., 2019), coffee (Kumar et al., 2020), grape (Liu et al., 2020), cassava (Ramcharan et al., 2017), tomato (Durmuş et al., 2017; Fuentes et al., 2017; Liu and Wang, 2020), and apple (Liu et al., 2017), as well as in multi-crops (Mohanty et al., 2016; Ferentinos, 2018; Too et al., 2018). In most cases, researchers fine-tune off-the-shelf Convolutional Neural Networks (CNNs) (Saleem et al., 2019).

Although the evaluated CNN methods in these publications appear to be effective and seem to learn relevant feature representations of the diseases, they unfortunately also learn irrelevant disease characteristics such as background noise (Mohanty et al., 2016; Atabay, 2017) or uninfected plant parts (Ferentinos, 2018; Toda and Okura, 2019; Lee et al., 2020). For example, (Atabay, 2017) has shown that a CNN trained on tomato plant diseases has neuron activations that fall mostly in the background. Unfortunately, it has been shown that background suppression with image segmentation does not give better results than an ordinary colored background with CNN (Mohanty et al., 2016), confirming a dependence of background characteristics for disease identification. Even worse, Ferentinos (2018) showed that a CNN tends to be confused between similar crops of different disease classes. It is thus indicated that a CNN model, which is supposed to learn the visual representation of plant diseases, tends to be biased toward irrelevant crop characteristics. Region-based deep neural networks can help to focus on contaminated parts (Fuentes et al., 2017, 2019), but such a technique involves labor-intensive annotations of disease locations and also depends heavily on prior knowledge of plant diseases.

**Figure 1:**
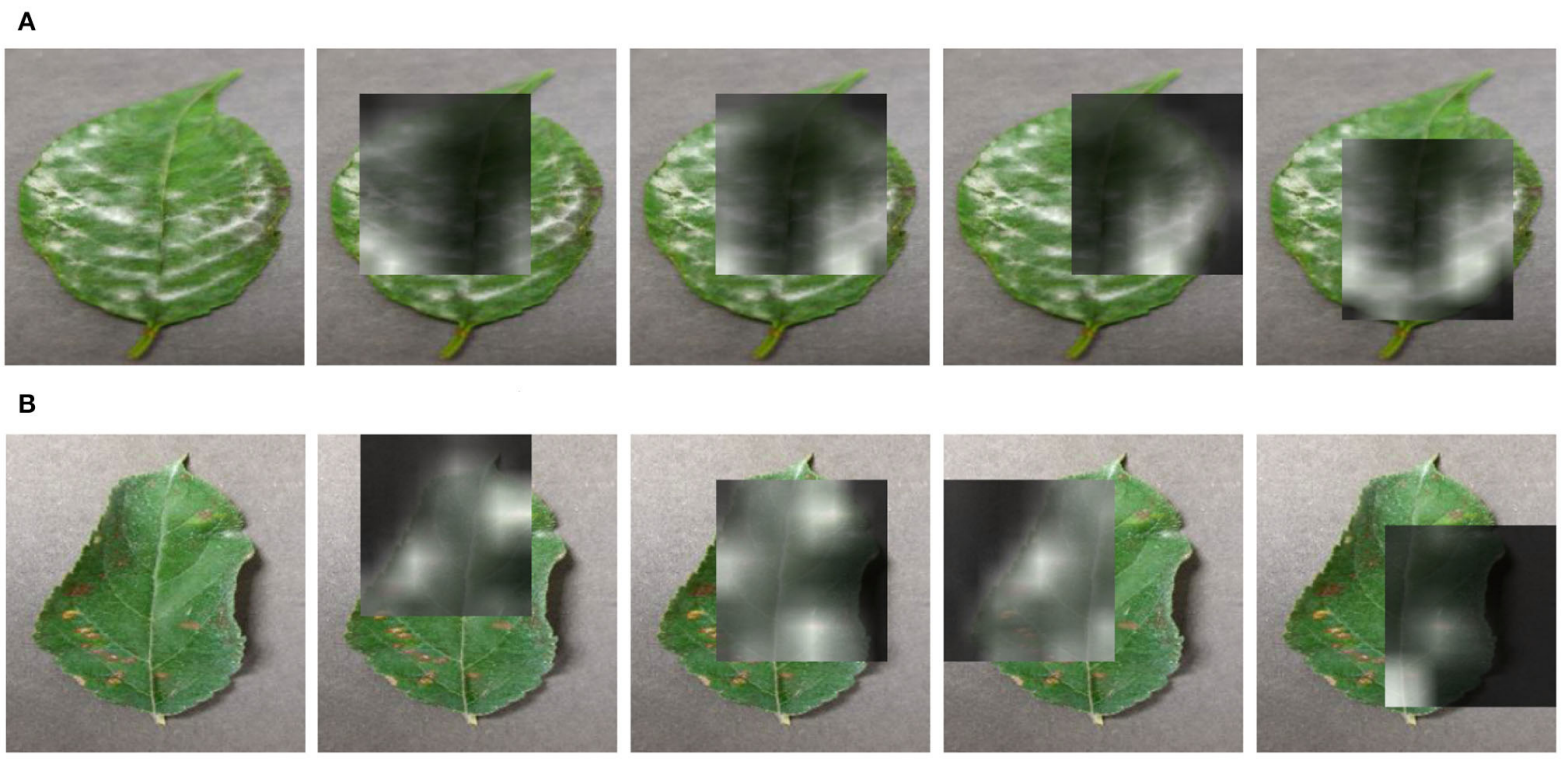
The learned attention maps by our proposed approach on two diseases that have contaminated leaves. The visualizations highlight the infected regions. **(A)** Powdery mildew disease, on cherry plant. **(B)** Cedar apple rust disease, on apple.

Henceforth, these observations have motivated us to go beyond existing practices by exploring a new technique for identifying plant diseases that allows us to automatically learn the regions of interest in the plant image, which correspond to the infected regions, and then to identify the diseases. Inspired by recent work on multi-organ plant identification that has shown the ability of an attention-based Recurrent Neural Network (RNN) to locate relevant regions of plant structures without any prior human annotation (Lee et al., 2018), we have adapted this approach to learn visual representations of plant diseases and show that discriminating infected regions of a plant can be successfully located and highlighted for disease identification, as illustrated in Figure 1.

Our contribution in this paper is three-fold: firstly, to our knowledge, this is the first time that the RNN-based approach is being explored to learn representations of plant diseases at that scale and that a comparison of identification performance is made against the widely used CNN approaches in this field. Second, we show quantitatively that the RNN approach outperforms the CNN approaches. Finally, we also show qualitatively that the RNN approach is able to detect precisely the infected regions through the neuron activations. The source code of our computational implementation is provided on an open online repository to facilitate its long-term accessibility and use by the scientific community^1^.

## 2. Materials and Methods

### 2.1. Attention-Based RNN Model

A recurrent neural network (RNN) is a class of neural networks where connections between nodes of a layer form a directed graph along a sequence of variables (e.g., a temporal sequence). The recurrent connections typically allow for modeling of the relationship between the current state of a variable and the previous states (similarly to a Markov chain). The RNN-based approach has received much attention because of its ability to handle sequential data to make predictions, such as in language translation (Sutskever et al., 2014) or action recognition (Du et al., 2018; Song et al., 2018). Improved RNN models, such as Long Short-Term Memory networks (LSTMs) or Gated Recurrent Units (GRU), enable training on long sequences, overcoming problems like vanishing gradients. Recently, a few publications have shown the effectiveness of RNN approaches to sequentially process variable-length data of fixed sizes, such as a picture. For example, it has been shown that a RNN architecture based on GRU can efficiently model dependencies between different images of plant observations Lee et al. (2018) or that LSTM can be used to capture discriminating regions of images for fine-grained classification (Zhao et al., 2017). Attention is a mechanism that can be combined in the RNN to allow it to focus on certain parts of the input when predicting a certain part of the output, thus enabling an easier learning and of higher quality. For instance, RNN with attention mechanism was used in Ren and Zemel (2017) to capture the spatial structure in images and produce detailed instance segmentation.

**Figure 2:**
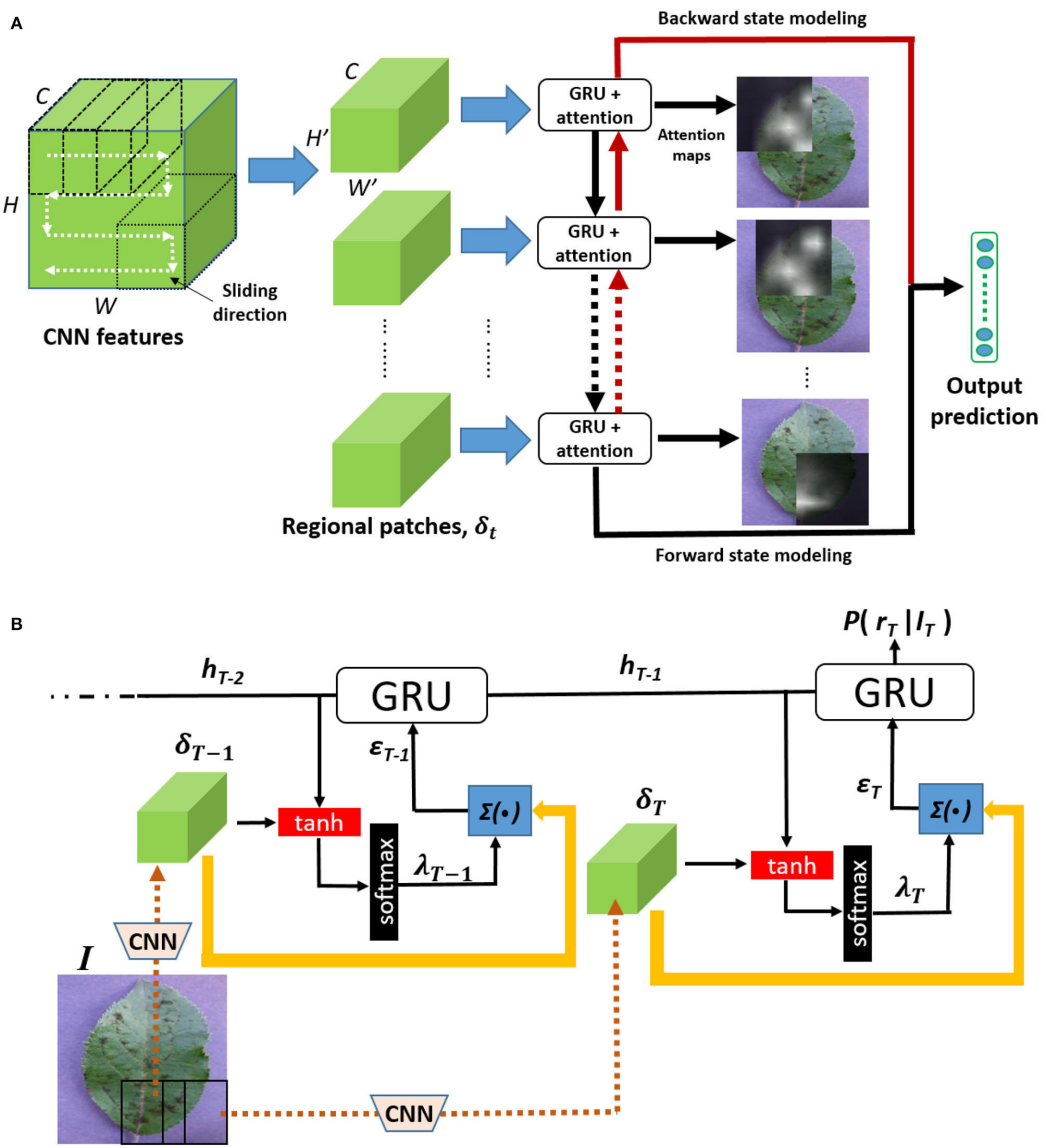
The proposed architecture: from an image of a contaminated plant, feature maps are first extracted from a given convolutional layer of a pre-trained CNN. They are then sliced into several patches following a “snaking” sliding direction. The patches then feed into Gated Recurrent Units that share, combine, and retain relevant information in a bidirectional way to update an internal representation of plant disease. Soft attention mechanism is used to infer discriminating local features. **(A)** Overall architecture. **(B)** Soft attention mechanism.

Inspired by these previous works, we combine in a RNN an attention mechanism with Gated Recurrent Units to dynamically push salient plant disease characteristics to the forefront in order to strengthen the model in learning disease characteristics for identification. Figure 2 shows the framework of the proposed architecture. First, a CNN trained on a plant disease classification task is used as a visual features extractor: a plant image is thus encoded as *CNN features* (i.e., a tensor of feature maps at a given output of a chosen convolutional layer). These CNN features can be considered as a new smaller image of activations with as many channels as filters used in the convolution layer. This new image is then sliced into sub-parts of the same size to get local activations in many regions covering all of the image. These new local CNN features can then be used to build a sequence and feed an RNN based on GRUs, enabling an attention mechanism to locate important parts or components in the CNN features. It extends the effective pixel neighborhood in each sub-part and maximizes the information gain across several sub-parts of the CNN features. Finally, prediction error is minimized throughout the optimization process.

#### 2.1.1. Our Overall RNN Architecture

We denote a plant disease image as **I** and the corresponding feature maps extracted by the convolutional layers of the CNN as δ ∈ ℝ^*H* × *W* × *C*^, where *H, W* and *C* are, respectively, the height, width, and number of channels in the feature maps. The CNN model is initially pre-trained and optimized purely based on plant disease target classes. A sequence of *T* regional feature maps {δ_1_, δ_2_, ⋯ , δ_*T*_} ∈ δ is then generated by slicing the global feature map δ following the sliding direction shown in Figure 2. The resulting sequence of feature maps is then used as input of the *RNN* module displayed in Figure 2A and detailed in Figure 2B. Thanks to the RNN connections between the different feature maps, the network is able to iteratively learn the discriminant visual patterns and to model the spatial relationship between them. For instance, brown specks that spread from side to side on a part of the leaf can be distinguished from brown specks that appear randomly on the leaf.

#### 2.1.2. Attention Module

The attention module is used to model the relative contribution of each pixel of the *T* regional feature maps. Specifically, it forces an explicit additional step in the reasoning process, identifying salient regions by assigning different importance to features from different image regions. The attention mechanism is introduced by the **λ_*t*_** terms (also called *regional attention map*) that control the contribution of the pixels of the *t*-th state and that are trained by the neural network. A larger **λ_*t*_** value indicates higher importance. More formally, the attention function *g*:δ_*t*_, **h_t−1_ → ϵ_*t*_** is defined as follows:

(1) $$\zeta_{t}=\{\tanh(\delta_{t}W_{\delta }+h_{t-1}W_{h})\}W_{a}$$

(2) $$\begin{array}{r} \lambda_{t}=softmax\left(\zeta_{t}\right) \end{array}$$

(3) $$\epsilon_{t}=g(\delta_{t},h_{t-1})=\sum_{i,j}\lambda_{t,ij}.\delta_{t,ij}$$

where $W_{\delta }\in {\mathbb{R}}^{C\times C}$, $W_{h}\in {\mathbb{R}}^{E\times C}$, $W_{a}\in {\mathbb{R}}^{C\times 1}$ are the embedding matrices, *E* is the dimensionality of GRU cell, and **δ_*t,ij*_** denotes the value of the *t*-th regional feature map at position (*i, j*) ∈ *H*′ × *W*′. Note that **ϵ_*t*_** is the output representation (feature vector) for the *t*-th regional feature map.

#### 2.1.3. Bi-Directional Training

Inspired by previous experiments (Lee et al., 2018) that show that bidirectional states modeling performs better compared to uni-state modeling in plant-view correlation learning, we built a bidirectional states modeling mechanism where the forward neuron activations $\vec{h_{T}}$ and the backward neuron activations $\overset{\leftarrow }{h_{0}}$ model *P*(**h_t_**|**δ_t_**, **h_0_**, ⋯ , **h_T−1_**) and *P*(**h_t_**|**δ_t_**, **h_T_**, ⋯ , **h_1_**), respectively. In order to correlate between both states, the final output activations of the forward and backward GRU are cascaded as follows: $h=[\vec{h_{T}},\overset{\leftarrow }{h_{0}}]$. We then multiply **h** with a class embedding matrix, **W_em_**, which is **s**(**I**) = **W_em_h** before normalizing it with a softmax function: $P\left(r|I\right)=\frac{e^{s_{r}\left(I\right)}}{\sum_{m=1}^{M}e^{s_{m}\left(I\right)}}$ where *M* and *r* stand for the total number of classes and the target class, respectively. After performing the softmax operation, we find the maximum likelihood of the sample by applying the objective function, *L* = −*logP*(*r*|**I**).

#### 2.1.4. Implementation Details

To extract the CNN features, we used an extension of a GoogleNet architecture Szegedy et al. (2015) with modified convolutional layers and additional batch normalization to increase accuracy and reduce computational complexity^2^. After training the CNN on the disease classification task, we extracted CNN features with a size of 14 × 14 × 576 from the convolutional layer *Inception_4d* and sliced them with a stride of 1 into regional patches with a size of *H*′ = *W*′ = 8 each to finally feed the RNN. The RNN is trained using the Tensorflow library (Abadi et al., 2016). We use the ADAM optimizer (Kingma and Ba, 2014) with the parameters α = 1*e* − 08, β1 = 0.9, and β2 = 0.999. We applied the weight decay *L*_2_ with the penalty multiplier set to 1 × 10^−4^ and dropout ratio set to 0.5, respectively. We set the learning rate to 1 × 10^−4^, and the mini batch size was set to 30.

#### 2.1.5. Features Visualization Method

**Figure 3:**
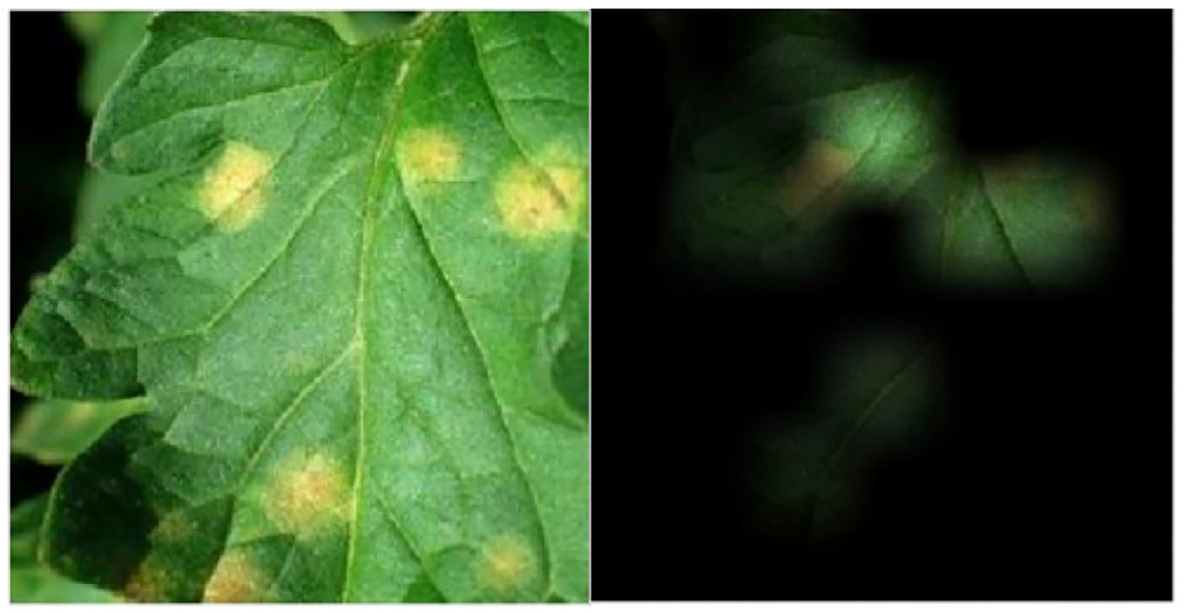
Visualization of activations of the learned features with the CNN model (GoogleNet). Best viewed in color.

**Figure 4:**
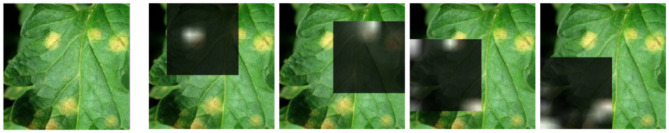
Visualization of attention maps learned using our attention-based RNN on a leaf infected by the *Leaf mold* disease (to be compared with Figure 3). Best view in color.

**Figure 5:**
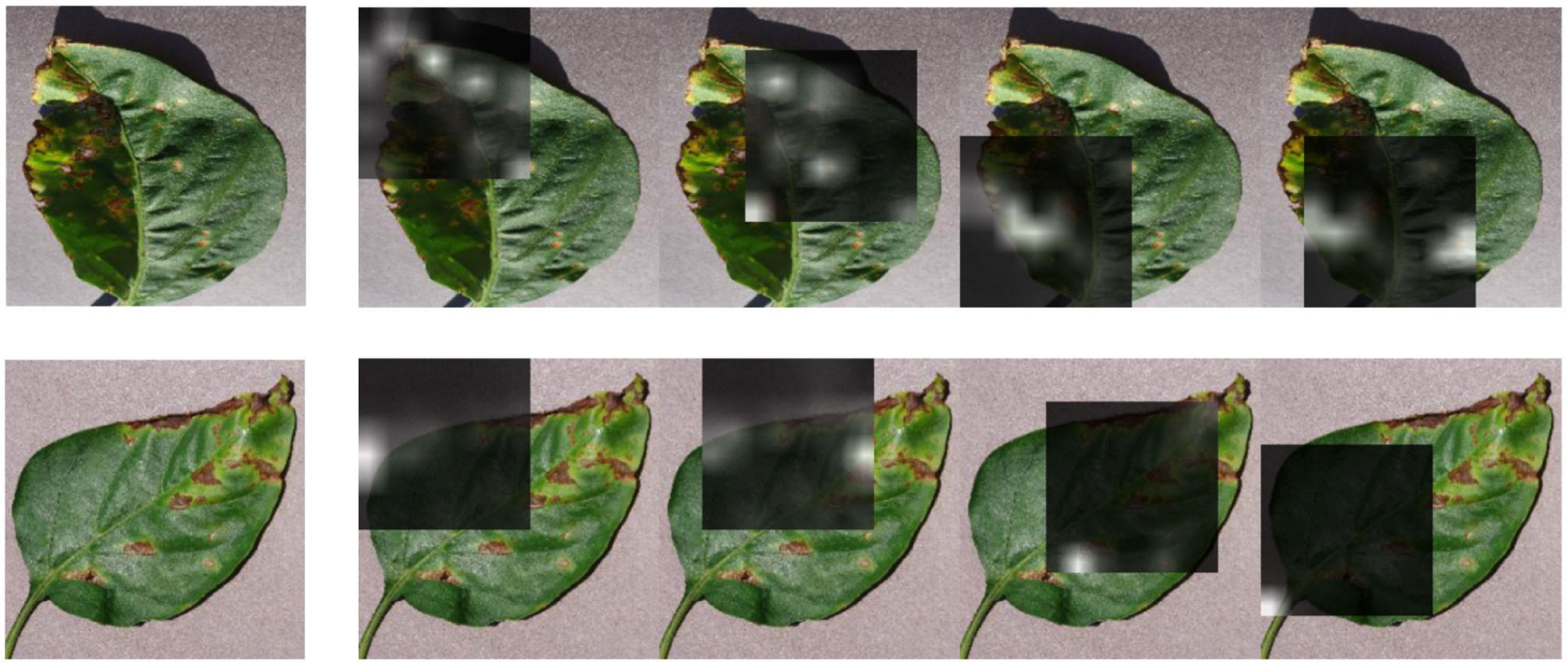
Visualization of attention maps learned using our new attention-based RNN model on two correctly classified images of the Unseen Crop of *pepper_bell Bacterial_spot*.

We describe here the methodology used to visualize the visual features captured by the CNN and the RNN model (see Figures 3–**5** of the results section). For the CNN model (GoogleNet), we first tracked the position of the highest activation across all the feature maps extracted from the last convolutional layer. From this, we accumulated the first 30 dominant activations and assessed them according to the original image. For the RNN model, we simply displayed a subset of the regional attention maps **λ_*t*_**.

### 2.2. Experimental Dataset

Plant Village (PV) (Hughes and Salathé, 2015) is a popular dataset dedicated to the evaluation of automated identification of plant diseases under controlled environments. It has 38 crop-disease pairs, with 26 crop-disease categories concerning 14 crop plants. The dataset was provided with predefined training and test sets, and a configuration with a percentage ratio of 80 and 20% is used, giving a total of 10,495 training images and 4,310 test images. Since plant diseases, named by vernacular names, share the same visual characteristics for different species, we categorized leaf samples from the PV dataset into 21 classes (20 diseases and one healthy class) and trained a classifier based on these classes. Note that, in practice, it is impossible to collect all disease samples from different crops under different environments to train a deep model. In fact, what we intended to achieve is a model that is general enough to represent knowledge in a way that can be transferred between different plant disease tasks. We therefore explored the generalization of models to identify disease of unseen crops and also plants that are captured under different contexts (typically in the field).

To evaluate the ability of the model to generalize to unseen crops, we excluded one crop from the training set and use it only for testing. More specifically, we removed all the leaf samples from the pepper crop, i.e., the ones from the *Pepper_bell Bacterial_spot* class and the ones from the *Pepper_bell healthy*, so as to treat the pepper class as an unseen crop in this experiment while the *Bacterial_spot* can be learnt through other crops. Besides the PV dataset, we also assessed the robustness of our models using pictures related to the same disease categorization and from reliable online sources that are not restricted to a controlled environment. We used 119 and 64 images from IPM and Bing, respectively, collected by Mohanty et al. (2016) (121 number of Bing images were expected, but half of them are no longer available).

## 3. Results and Discussion

The experimental results are presented in the Table 1. As there are only few images of pepper crops in IPM and Bing (two images in IPM and one in Bing), we considered the number of collections insufficient to infer the performance of the models in recognizing the disease of an unseen crop. We therefore tested the model using only the seen crop images in IPM and Bing. We compared the performance of the models with the Inception-V3 model employed by Brahimi et al. (2018) since it was reported to be the best approach for disease identification on the PV dataset. The model is trained on the basis of the aforementioned 21 target classes with the pepper crop excluded from the training set.

**Table 1:** Top-1 accuracy (%) comparison on Plant Village (PV), IPM, and Bing.

**Method**	**PV-SC**	**PV-UCB**	**IPM-SC**	**Bing-SC**
CNN InceptionV3 of Brahimi et al. (2018)	98.05	29.63	29.06	28.57
Our CNN (GoogleNet)	**99.17**	18.98	37.61	36.51
Our new model Seq-RNN	98.17	**58.80**	**40.17**	**39.68**

*Note that the performance of IPM and Bing are based only on Seen Crop (SC) images, whereas the performance on PV is measured for both Seen Crop (PV-SC) and Unseen Crop (PV-UCB), i.e., for the pepper_bell Bacterial_spot*.

*The best accuracy for each dataset is shown in bold*.

First, by examining the accuracy of the PV-SC test set (seen crops from the PlantVillage dataset), we can see that both CNN models and our new attention-based RNN achieved very high accuracy values. This is mainly due to the fact that the images in this test set were acquired under exactly the same conditions as the training set so that any method can exhibit a high performance. Secondly, we can observe that both CNN models as well as our new attention-based RNN achieved lower accuracy values for the IPM-SC and Bing-SC test sets as well as the PV-UCB (unseen crop from the PlantVillage dataset) compared to the PV-SC. We believe that this is due to a change in the data distribution between the PV data and the IPM-SC and BING-SC test sets, as the two data are collected under different conditions, and the disease training data are not sufficiently diverse to cover the ranges of visual appearance of the disease found in the unseen crop. To cope with such difficult datasets, CNN models achieved a much lower performance, which means that the models formed have some difficulty in generalizing to the unseen crop of PlantVillage (PV-USC) or to images acquired in a different domain (IPM-SC and Bing-SC). In comparison, our attention-based RNN model was much more accurate over the three series of tests. Although the accuracy is far from perfect, it is still quite reasonable considering the difficulty of the problem.

We further compared the generalization ability of our new model and that of a classical CNN thanks to visualization experiments presented in Figures 3, 4. As shown in Figure 3, the regions of the image leading to a strong activation of the CNN neurons do not really seem to correspond to the visual patterns characteristic of the disease. Indeed, they largely correspond to healthy leaf features, specifically the venation. On the contrary, Figure 4 shows that the activated regions of the attention maps of our new RNN model do match the disease spots much more precisely and accurately. Figure 5 shows a similar visualization on two leaves of the *Pepper_bell* crop affected by *Bacterial_spot*. Here again, we can observe that most of the activated regions do correspond to altered parts of the leaf, whereas the *Pepper_bell* was not even present in the training set.

In line with our results, we deduced that the transferability of Seq-RNN knowledge is more useful than that of CNN in differentiating data, especially those taken in the field. This could be due to the fact that CNN is learned on the basis that its spatial information collapses in the final convolutional layer, resulting in the relativity of local features that are important for representing diseases not retained. This might be also the reason the CNN searches for global characteristics that are not relevant for the infected area but are relevant for leaf characteristics, such as shape and venation.

On the other hand, Seq-RNN takes the convolutional map, where substantial spatial information is retained as input and is formulated in such a way as to learn the relationship between the neighboring regions of an image. Because of this formulation, the Seq-RNN's attention mechanism will be forced to detect salient regions, where, in this case, there are the obvious infected regions that appear in the local areas of an image. This disease-focused knowledge could be effectively transferred to field images where the plant structure may not be easily visible (due to organ deformation or clutter) but the visual aspect of the disease is evident. Thus, in field images where the plant structure is not visible or may be difficult to distinguish, the disease characteristics become more determinant, allowing Seq-RNN, which has a greater knowledge of the disease, to better differentiate the data than the CNN.

## 4. Conclusions and Future Directions

We presented in this study a new efficient computational architecture that opens up new perspectives for the automated classification of plant diseases. We showed that our RNN approach has a higher generalization ability than the classical CNN approach, especially in distinguishing disease samples that are different from the training set. This is a major critical point in plant pathology, as it is highly difficult (due to required expertise level and time consumed) to produce a complete and diversified visual dataset of all the symptoms of any crop disease at a global scale. Our approach could thus overcome the problems related to the lack of training data usually necessary for the development of performing recognition deep learning models.

In this study we also analyzed the attention maps learned by our RNN and showed that it meets our expectation of localization of infectious diseases in plants images. We believe that our RNN-based approach, which captures the context of the relationship between local features, could provide a better insight into where the machine actually detects relevant information. It is important to note that by assessing the machine's perspective in disease differentiation, humans will certainly benefit, as we all know that human capabilities are limited when it comes to identifying the thousands of diseases for all the plants in the world.

In this paper, we have shown that the integration of our RNN approach in the design of the deep learning architecture for learning disease representation can not only provide a better classification performance but also contribute to the knowledge of plant diseases, which could potentially be useful to address doubts that have not yet been resolved. Future research directions should focus on the integration of CNN and RNN based models to simultaneously address the learning of rich global and local visual representations within a deep end-to-end network. Furthermore, alternative slicing patterns on convolutional maps before transmitting the extracted patches to RNN should be studied for a better modeling of the local characteristics of the plant disease. In addition, complementary statistical analyses should be performed on the evaluation of the RNN approach for field images that are of main interest to farmers and all field agricultural actors. We believe that these findings could encourage future research to rethink the current de facto paradigm of purely relying on the CNN in plant disease identification.

## Data Availability Statement

Publicly available datasets were analyzed in this study. This data can be found here: https://doi.org/10.3389/fpls.2016.01419 (Mohanty et al., 2016) and https://arxiv.org/abs/1511.08060 (Hughes and Salathé, 2015).

## Author Contributions

SL and HG conducted experiments and data analysis. SL wrote the first draft of the manuscript. HG, PB, and AJ revised the manuscript. All authors designed the research, contributed to the article and approved the submitted version.

## Conflict of Interest

The authors declare that the research was conducted in the absence of any commercial or financial relationships that could be construed as a potential conflict of interest.

## Footnotes

^1^the web link to the open online repository on which the source code is hosted will be displayed here after acceptance of the manuscript.

^2^https://github.com/AdelineMomo/CNN-plant-disease (Caffe).
